# Supplementary material for: Viral proteins as a potential driver of histone depletion in dinoflagellates
Source: Nat Commun. 2018 Apr 18;9:1535. doi: 10.1038/s41467-018-03993-4 (PMC5906630; doi:10.1038/s41467-018-03993-4)
Supplement: Supplementary file 1 — Supplementary Information [file 41467_2018_3993_MOESM1_ESM.pdf]

Supplementary Figures and Tables

Viral proteins as a potential driver of histone depletion in dinoflagellates

Irwin et al.

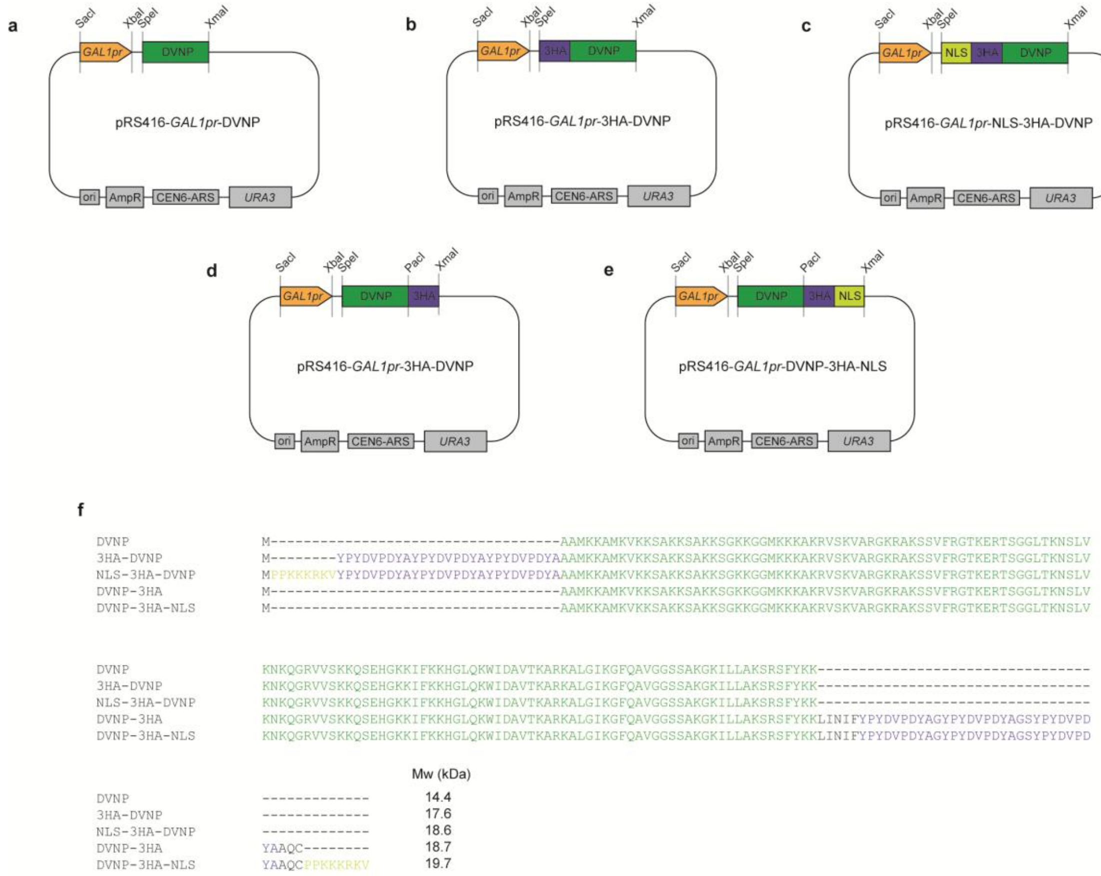

Supplementary Figure 1 | DVNP expression vectors. a-e, Schematic representations of expression vectors used in this study. *GAL1pr*, *GAL1* promoter; 3HA, three hemaglutinin epitope tags; NLS, nuclear localization signal; ori, bacterial origin of replication, AmpR, ampicillin resistance gene; CEN6-ARS, centromeric yeast autonomous replicating sequence. **f,** Amino acid sequences of all DVNP constructs used in this study. Molecular weights for each construct are shown. DVNP, 3HA tags, and NLS are shown in green, blue, and yellow, respectively.

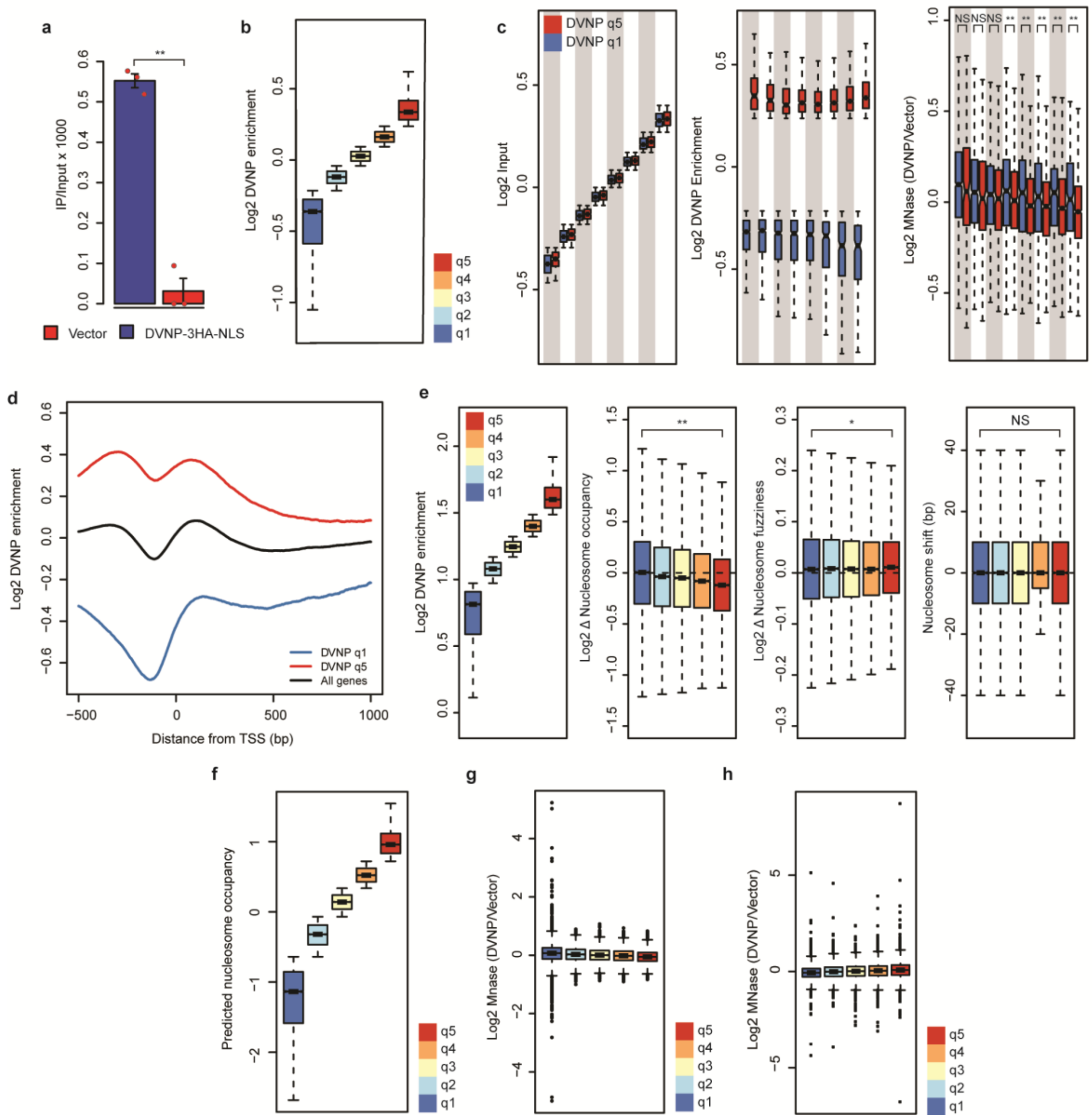

Supplementary Figure 2 | Supplemental information for DVNP ChIP sequencing and MNase sequencing. a, ChIP efficiency from DVNP-3HA-NLS-expressing and vector control cells (n = 3 biological replicates). Error bars represent SEM. This experiment was repeated twice with the same results. **b,** DVNP enrichment within the 20% DVNP quintiles shown in Fig. 2c. **c,** Genome wide association between DVNP enrichment and differential nucleosome occupancy in 10% input quintiles. The 2nd to 9th input quintiles (first panel) were subdivided into top and bottom 20% DVNP quintiles (q1,5, second panel) (n = 966). The ratio between DVNP-3HA-NLS and control MNase occupancy (third panel) is shown in these bins. **d,** DVNP enrichment within the 20% DVNP quintiles shown in Fig. 2d. **e,** DANPOS calculated changes in nucleosome occupancy (2nd panel), fuzziness (3rd panel), and position (4th panel), binned into 20% DVNP quintiles (q1-q5, 1st panel) (n = 14557). **f,** Predicted nucleosome occupancy within the 20% quintiles shown in Fig. 2e. **g-h,** Fig. 2c and Fig. 2e showing outliers. *P*-values were obtained by two sided Welch's t-tests. Box plot wedges represent an estimate of the 95% confidence interval of the median. *, $P < 0.05$; **, $P < 10^{-3}$; NS, not significant.

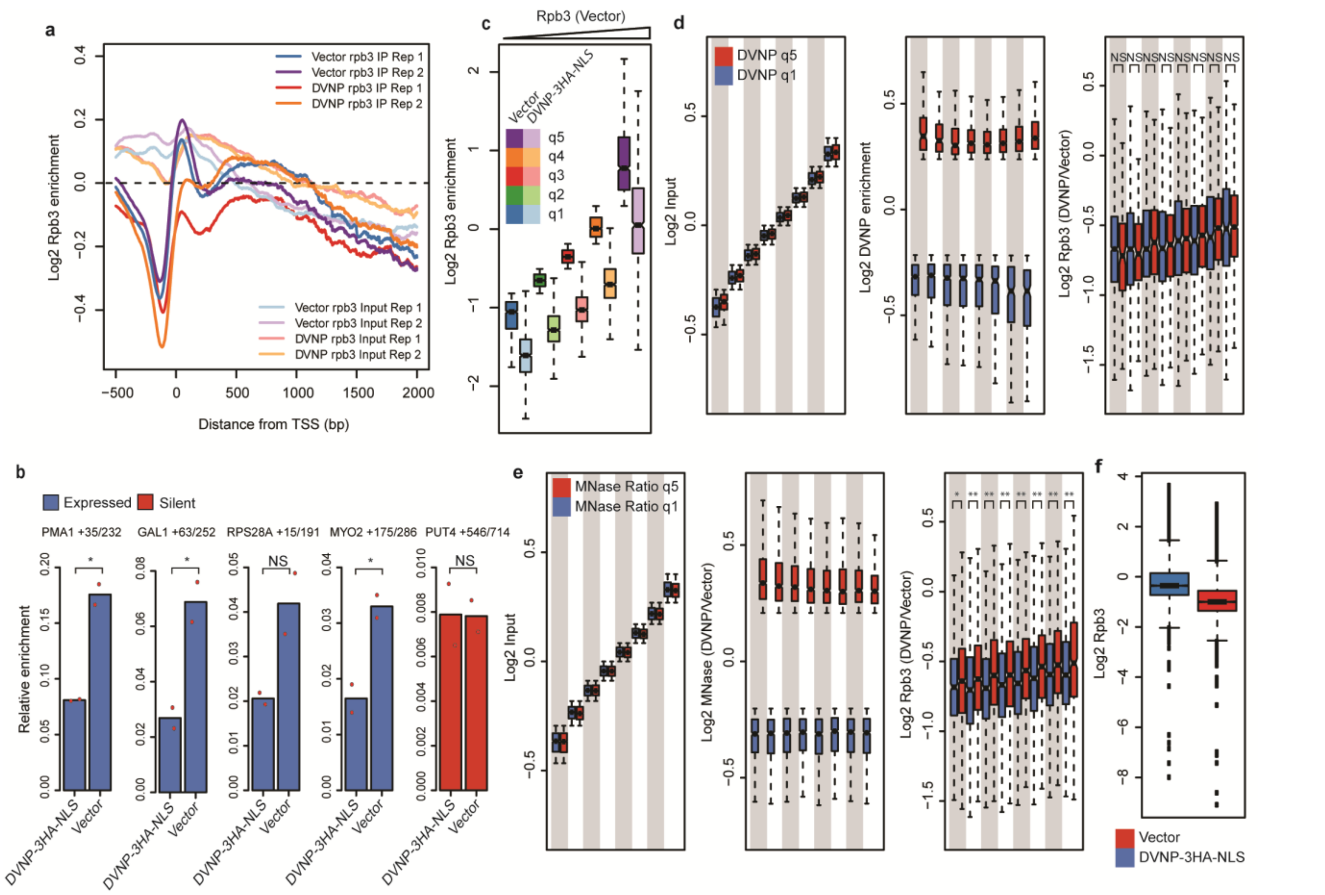

Supplementary Figure 3 | Supplemental information for Rpb3 ChIP sequencing. a, Average gene plot showing the relative enrichment of Rpb3 chromatin immunoprecipitates (ChIPs), and inputs from DVNP-3HA-NLS-expressing and control cells without adjusting for spike-in controls. Two biological replicates are shown for the ChIPs and inputs. **b,** Rpb3 ChIP-qPCR enrichment at five loci in DVNP-3HA-NLS expressing and control cells (n = 2 biological replicates). Expressed and silent genes are shown in red and blue, respectively. Values were made relative by normalizing to an arbitrary input. **c,** Rpb3 loss and transcriptional state are not associated. Genes were binned into 20% vector Rpb3 ChIP quintiles to reflect transcriptional level. Rpb3 enrichment from DVNP-3HA-NLS-expressing (faded colours) and control cells (bold colours) are plotted in these bins. **d-e,** Genome wide association between DVNP enrichment, differential nucleosome occupancy, and Rpb3 loss in 10% input quintiles. The 2nd to 9th input quintiles (first panels) were subdivided into top and bottom 20% DVNP quintiles (d) or MNase ratio quintiles (e, second panels). The change in Rpb3 is shown in these bins (third panels) (n = 966). **f,** Fig. 3b showing outliers. *P*-values were obtained by two sided Welch's t-tests. Box plot wedges represent an estimate of the 95% confidence interval of the median. *, $P < 0.05$; **, $P < 10^{-5}$; NS, not significant.

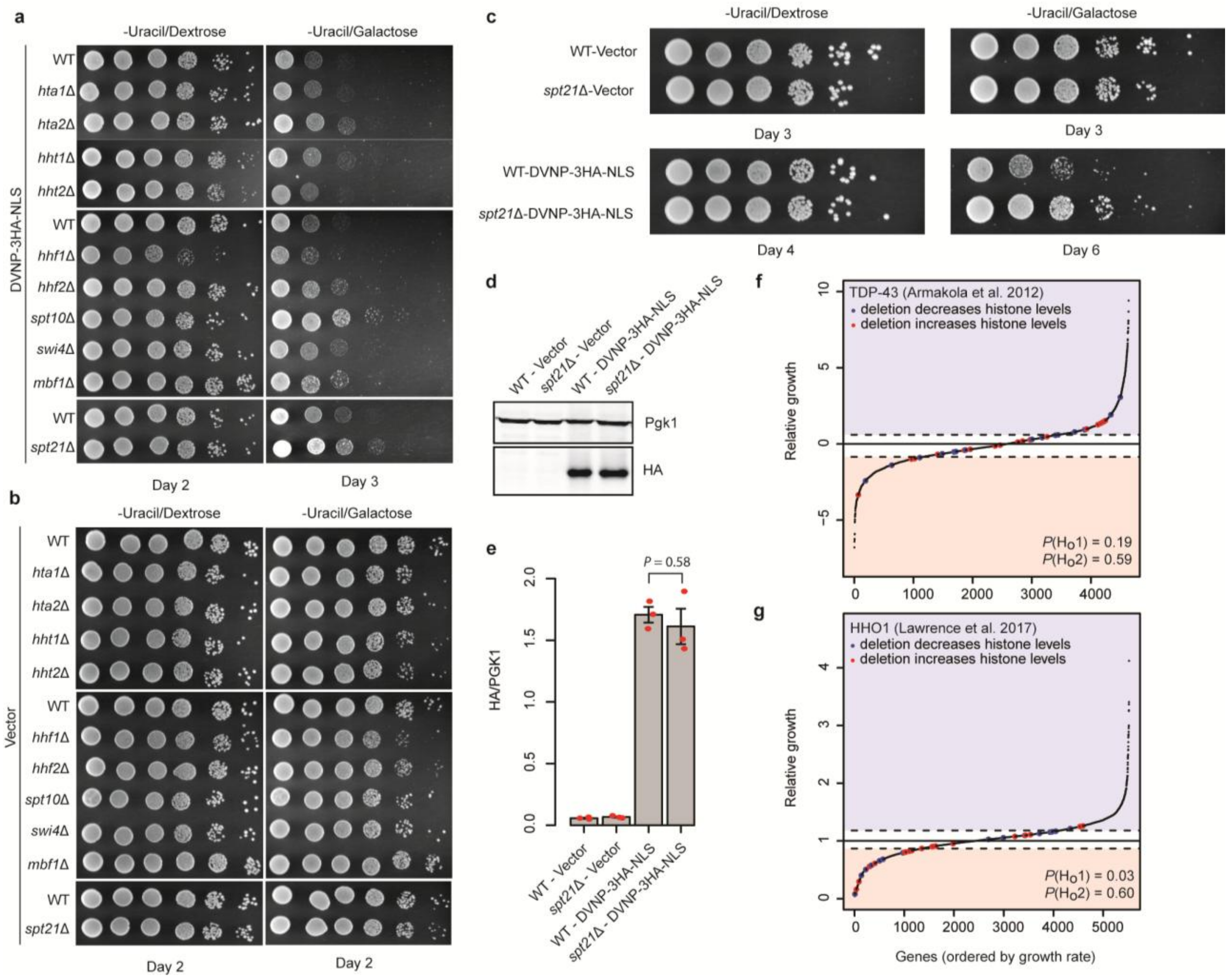

Supplementary Figure 4 | Supplemental information for the SGA. a-b, Serial dilution growth assays for DVNP-3HA-NLS-expressing (**a**) and control cells (**b**). Histone and histone activator mutants from the SGA screen were obtained and freshly transformed with constructs prior to assessing growth. Cells were grown on selective media lacking uracil in the presence of either dextrose or galactose. This experiment was repeated twice with the same results. **c,** Serial dilution growth assays for DVNP-3HA-NLS expressing and control cells in a wildtype (WT) and newly generated *spt21*Δ mutant in a different strain background than the deletion collection. Cells were grown on selective media lacking uracil in the presence of either dextrose or galactose. This experiment was repeated three times with the same result. **d,** Immunoblot on total protein extracts following galactose induction. **e,** Quantified immunoblot data showing similar levels of HA (DVNP) in the WT and newly generated *spt21*Δ strain (n = 3 biological replicates). Error bars represent the SEM. The *P*-value was obtained by Welch's t-test. This experiment was repeated twice with similar results. **f-g,** Previously published data showing the relative growth of ~4500 non-essential deletion strains expressing TDP-43 (**f**) or *HHO1* (**g**) from a *GAL1* promoter. Gene deletions known to increase and decrease histone expression are shown in red and blue, respectively. Two null hypotheses were tested by χ^2 test: H_o1: gene deletions that affect histone levels are randomly distributed and, H_o2: gene deletions that increase and decrease histones are enriched below and above the growth thresholds by chance. Dashed lines denote positive and negative growth thresholds.

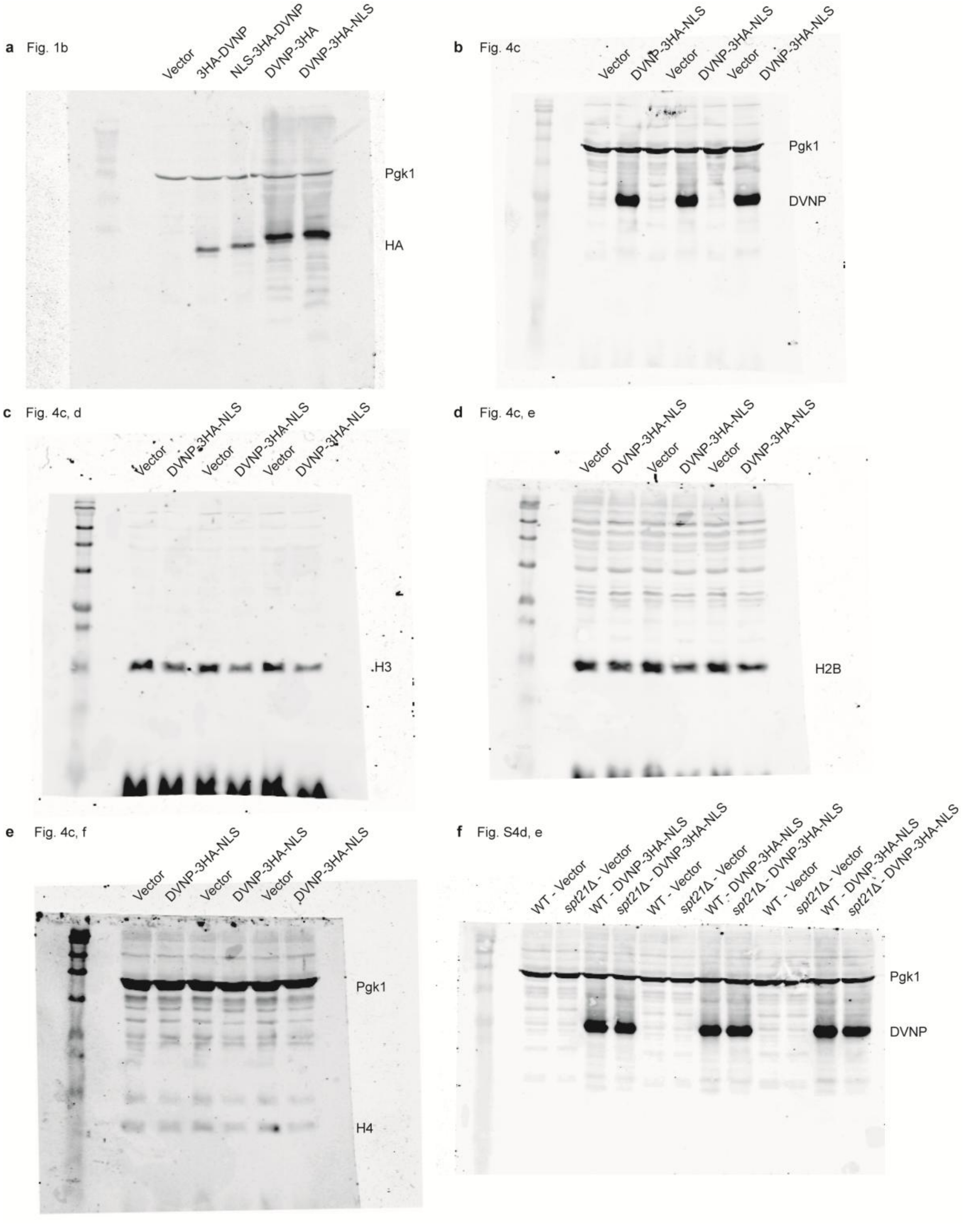

Supplementary Figure 5 | Uncropped images of the immunoblots shown in the indicated figures.

Supplementary Table 1. Yeast strains used in this study

Strain name	Mating type	Genotype	Reference
FY602	Mat a	*his3 Δ200 leu2 Δ1 lys2-128δ ura3-52 trp1 Δ63*	Sterner et al.[1]
Y7092	Mat α	*can1Δ::STE2pr-Sp_his5 lyp1Δ his3 Δ1 leu2 Δ0 ura3 Δ0 met15 Δ0 trp1::NATMX*	Tong & Boone[2]
BY4730	Mat a	*his3 Δ1 leu2 Δ0 met15 Δ0 ura3 Δ0*	Winzeler et al.[3]
hta1Δ	Mat a	*his3 Δ1 leu2 Δ0 met15 Δ0 ura3 Δ0 hta1::KANMX*	Winzeler et al. [3]
hta2Δ	Mat a	*his3 Δ1 leu2 Δ0 met15 Δ0 ura3 Δ0 hta2::KANMX*	Winzeler et al. [3]
hht1Δ	Mat a	*his3 Δ1 leu2 Δ0 met15 Δ0 ura3 Δ0 hht1::KANMX*	Winzeler et al. [3]
hht2Δ	Mat a	*his3 Δ1 leu2 Δ0 met15 Δ0 ura3 Δ0 hht2::KANMX*	Winzeler et al. [3]
hhf1Δ	Mat a	*his3 Δ1 leu2 Δ0 met15 Δ0 ura3 Δ0 hhf1::KANMX*	Winzeler et al. [3]
hhf2Δ	Mat a	*his3 Δ1 leu2 Δ0 met15 Δ0 ura3 Δ0 hhf2::KANMX*	Winzeler et al. [3]
spt10Δ	Mat a	*his3 Δ1 leu2 Δ0 met15 Δ0 ura3 Δ0 spt10::KANMX*	Winzeler et al. [3]
spt21Δ	Mat a	*his3 Δ1 leu2 Δ0 met15 Δ0 ura3 Δ0 spt21::KANMX*	Winzeler et al. [3]
swi4Δ	Mat a	*his3 Δ1 leu2 Δ0 met15 Δ0 ura3 Δ0 swi4::KANMX*	Winzeler et al. [3]
mbf1Δ	Mat a	*his3 Δ1 leu2 Δ0 met15 Δ0 ura3 Δ0 mbf1::KANMX*	Winzeler et al. [3]
FY602-*spt21Δ*	Mat a	*his3 Δ200 leu2 Δ1 lys2-128δ ura3-52 trp1 Δ63 spt21::HIS3MX6*	This study

Supplementary Table 2. Spike-in control sequences.

Spike-in	Sequence
**1**	TAACTCTTTCGCTCCCTCATTCGTTCCTTCGCTAGTACCAGACCAACTGGTAATG GTAGCGACCGGCGCTCAGCTGGAATTCCGCCGATACTGACGGGCTCCAGGAGTC GTCGCCACCAATCCCCATATGGAAACCGTCGATATTCAGCCATGTGCCTTCTTCC GCGTGCAGCAG
**2**	TAACTCTTTCGCTCCCTCATTCGTTCCTTCGCTAGTGCCGCCTTCATACTGCACCG GGCGGGAAGGATCGACAGATTTGATCCAGCGATACAGCGCGTCGTGATTAGCG CCGTGGCCTGATTCATTCCCCAGCGACCAGATGATC

Supplementary Table 3. qPCR primers used in this study.

Primer Name	Sequence
PMA1 +85	CTTACGATGACGCTGCATC
PMA1 +232	CCTCTGGAACTGGTCTAGC
GAL1 +63	CCAAGACCATTGGCCGAAAAG
GAL1 +252	CAAAACTTTGACGGCGCAAAGC
RPS28A +15	CCAGTCACTTTAGCCAAGGTC
RPS28A +191	CGAGCTTCACGTTCAGATTCC
MYO2 +175	CTCTACCGCTTCTTAGAAACCCTC
MYO2 +286	GAGAATAGCGCTGTTTGATGGC
PUT4 +546	CTGGTCACTAGGTACGTTGAC
PUT4 +714	CACGCATAGAAAGATCGTGATCC

Supplementary References

1. Sterner, D. E. *et al.* Functional Organization of the Yeast SAGA Complex : Distinct Components Involved in Structural Integrity , Nucleosome Acetylation , and TATA-Binding Protein Interaction. *Mol. Cell. Biol.* **19,** 86–98 (1999).

2. Tong, A. H. Y. & Boone, C. Synthetic genetic array analysis in *Saccharomyces cerevisiae*. *Methods Mol. Biol.* **313,** 171–92 (2006).

3. Winzeler, E. A. *et al.* Functional Characterization of the *S* . *cerevisiae* Genome by Gene Deletion and Parallel Analysis. *Science* **285,** 901–907 (1999).
