# Supplementary material for: Viral proteins as a potential driver of histone depletion in dinoflagellates
Source: Nat Commun. 2018 Apr 18;9:1535. doi: 10.1038/s41467-018-03993-4 (PMC5906630; doi:10.1038/s41467-018-03993-4)
Supplement: Supplementary file 3 — Description of Additional Supplementary Files [file 41467_2018_3993_MOESM3_ESM.pdf]

**Description of Additional Supplementary Files**

**File Name:** Supplementary Data 1
**Description:** DVNP synthetic genetic array data
